# Uric Acid—An Emergent Risk Marker for Thrombosis?

**DOI:** 10.3390/jcm10102062

**Published:** 2021-05-12

**Authors:** Laura Țăpoi, Delia Lidia Șalaru, Radu Sascău, Cristian Stătescu

**Affiliations:** 1 Institute of Cardiovascular Diseases, 700503 Iasi, Romania; laura.tapoi@yahoo.com (L.Ț.); radu.sascau@gmail.com (R.S.); cstatescu@gmail.com (C.S.); 2 Faculty of Medicine, University of Medicine and Pharmacy Grigore T.Popa, 700115 Iași, Romania

**Keywords:** uric acid, venous thromboembolism, atrial thrombosis, coronary thrombosis

## Abstract

Hyperuricemia is nowadays an established cardiovascular risk factor. Experimental studies linked elevated serum uric acid (SUA) levels with endothelial dysfunction (ED), inflammation, and prothrombotic state. The purpose of this review is to summarize the current evidence that emphasizes the possible role of uric acid as a biomarker for a prothrombotic state. A large number of clinical trials correlated SUA levels with both incident and recurrent cases of venous thromboembolism (VTE), independent of other confounding risk factors. Moreover, increased SUA levels may be an important tool for the risk stratification of patients with pulmonary embolism (PE). Left atrial thrombosis was correlated with high SUA levels in several studies and its addition to classical risk scores improved their predictive abilities. In patients with acute myocardial infarction (MI), hyperuricemia was associated with increased mortality, and the idea that hyperuricemia may be able to act as a surrogate to unstable coronary plaques was advanced. Finally, SUA was correlated with an increased risk of thromboembolic events in different systemic diseases. In conclusion, uric acid has been considered a marker of a thrombotic milieu in several clinical scenarios. However, this causality is still controversial, and more experimental and clinical data is needed.

## 1. Introduction

Hyperuricemia, a condition with an increased prevalence worldwide, has been associated with gout, type 2 diabetes mellitus, hypertension, and cardiovascular disease (CVD). Mendelian randomization (MR) studies showed significant correlations between hyperuricemia and coronary heart disease, peripheral artery disease [1,2,3], and atrial fibrillation (AF) [4]. In addition, uric acid was significantly associated with the risk of both cardiovascular and sudden cardiac death [2]. On the other hand, there are several MR studies that did not find causal associations between increased SUA and MI, blood pressure, coronary heart disease [5], or heart failure [6]. Moreover, it has been suggested that hypertension mediates the effect of hyperuricemia on CVD risk and that urate-lowering treatment may decrease blood pressure and the occurrence of major adverse cardiovascular events (MACE) in patients with pre-existing CVD [3]. The physiopathological relationship between hyperuricemia and CVD remains controversial and is still a matter of research. Evidence promotes links between elevated SUA levels and ED, inflammation, and prothrombotic state [7].

## 2. Mechanistic Framework

Uric acid is the end product of purine metabolism catalyzed by xanthine oxidase (XO). In the process of purine metabolism, reactive oxygen species (ROS), including superoxide, are generated concomitantly with uric acid production. Excessively generated superoxide anion (O2−) concomitant with increased uric acid production reacts directly with nitric oxide (NO) with high affinity, resulting not only in decreased NO bioavailability through degradation and inactivation of NO but also in increased formation of peroxynitrite (ONOO−), a highly potent oxidant causing deoxyribonucleic acid (DNA) damage, cell death, and lipid peroxidation. Therefore, it is postulated that bioavailability of NO is decreased by generated O_2_—concomitant with uric acid production in the process of purine metabolism catalyzed by XO, leading to ED [8]. It is speculated that extracellular uric acid has antioxidant properties by effectively scavenging free radicals in human plasma, but this benefit might be disturbed by the hydrophobic lipid layer of the cell membrane. In contrast, intracellular free oxygen radicals are produced during uric acid degradation and superoxide is further enhanced by interacting with nicotinamide adenine dinucleotide phosphate (NADPH) oxidase [9]. The physiopathological links between uric acid and CVD have been extensively discussed elsewhere [10,11]. Thus, we will only reiterate the main features that support the relationship between uric acid and the prothrombotic state.

It is hypothesized that uric acid induces vascular damage by several mechanisms. The first one is mediated by ROS, resulting in inflammation of the vascular endothelial cells and proliferation of vascular smooth muscle cells [12]. Previous studies reported that oxidative stress accompanied by the generation of ROS can be observed in cells exposed to uric acid [13,14]. Moreover, high level of uric acid could induce oxidative stress and inhibit cardiomyocytes viability through the ERK/P38 pathway [15]. Inflammation interferes with the coagulation system, by promoting clotting and by decreasing the activity of anticoagulation mechanisms, suppressing fibrinolysis and generating ED. Inflammatory states are significant risk factors for both the progression of atherosclerosis and thrombus formation in the venous system [16]. Increased SUA levels were associated with C-reactive protein (CRP), interleukin (IL)-6, inflammatory cytokines, and tumor necrosis factor-α (TNF-α) [17]. In addition, uric acid itself, even at normal concentrations, has proinflammatory consequences on vascular cells. It can trigger the production of the nucleotide-binding domain, leucine-rich-containing family, pyrin domain-containing-3 (NLRP3) inflammasome, which enhances the production of IL-1Ꞵ and augments the inflammatory response on vascular cells [18]. Moreover, activated endothelial cells are characterized by an increased expression of cell adhesion molecules. When ED is present, endothelin-1 and platelet activating factor promote vasoconstriction, while tissue factor (TF), plasminogen activator inhibitor-1 (PAI-1), von Willebrand factor, and Factor V enhance thrombosis [19].

**Figure 1:**
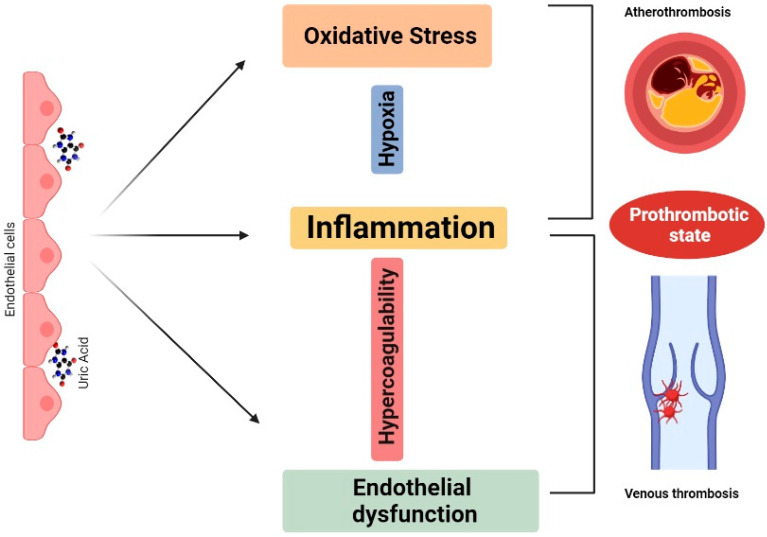
Proposed mechanism for the implication of uric acid in thrombosis. Once absorbed in the endothelial cells, uric acid causes inflammation, oxidative stress, and endothelial dysfunction. Inflammation, oxidative stress, and plaque hypoxia are shared mechanisms of atherothrombosis, while inflammation and endothelial injury in the context of hypercoagulability leads to venous thrombosis.

The second mechanism is mainly driven by vascular endothelium injury, which is one of the initial triggers incriminated in clotting formation. Preliminary studies suggested that secondary to its absorption into endothelial cells, uric acid may cause oxidative stress, inflammation, and dephosphorylation of endothelial nitric oxide synthase (eNOS), leading to ED through decreased NO bioavailability. In the context of hypercoagulability, inflammation and endothelial injury may promote venous thrombosis, while in the context of plaque hypoxia, inflammation and oxidative stress may lead to atherothrombosis [8] (Figure 1).

Despite numerous confounding factors, previous in vivo studies have demonstrated that elevated uric acid level was significantly associated with ED assessed by different endothelial function testing (e.g., flow-mediated dilation, reactive hyperemia index, and intracoronary acetylcholine testing) in various populations [10,20,21]. However, it has not been entirely elucidated whether uric acid per se is an independent causal risk factor of ED in humans. Although experimental studies have indicated that uric acid causes ED through increased oxidative stress and inflammation [8], an actual biological effect of uric acid on endothelial function in vivo has not been fully elucidated.

Furthermore, uric acid may indirectly induce prothrombotic status through its association with cardiovascular comorbidities. For example, uric acid has been correlated with left ventricular diastolic dysfunction, which in turn is characterized by increased filling pressures, resulting in low velocity of the blood stream, left atrial stretching, and maintenance of AF, all these exacerbating blood stasis. Moreover, inflammation promotes left atrial remodeling [22].

Increased uric acid is involved in the upregulation of lethal 7-c (let 7-c). Moreover, let 7-c is interconnected with platelets functionality. In a study performed on an animal model of hyperuricemia, increased levels of SUA generated thrombosis through the activation of MEF2C-dependent and NF-ƙB pathways by let 7-c [23]. In mice, the inhibition of xanthine oxidase by febuxostat led to a decrease in the expression of PAI-1 and TF, thus diminishing the prothrombotic state [24].

Platelet-derived microparticles (PDMPs) are prothrombotic molecules that contribute to thrombus formation and can therefore determine vascular complications. Increased levels of PDMPs may promote hypercoagulable states. Febuxostat therapy led to a significant reduction in PDMP levels in 69 hyperuricemic patients and decreased the expression of sP-selectin, thus being a potentially beneficial drug for the prevention of athero-thrombotic events [25]. On the other hand, in a study that included 493 patients on dual antiplatelet therapy, SUA levels were not correlated with high-residual platelet reactivity, thus concluding that uric acid did not affect the response to antiplatelet therapy [26].

Finally, uric acid may act as an intermediary between atherosclerosis and VTE. In a study that included 276 patients with idiopathic VTE and 536 controls, SUA was significantly associated with thrombotic events only in those with high HDL-C values [27].

However, is uric acid really in the front line of a prothrombotic state?

## 3. Uric Acid as a Risk Marker for Thrombosis in Different Clinical Scenarios

### 3.1. Uric Acid and VTE

VTE is causing considerable morbidity and mortality and therefore a comprehensive risk stratification is of utmost importance.

The Atherosclerosis Risk in Communities (ARIC) Study was the first to evaluate the relationship between SUA and VTE incidence. At baseline, there was measured a series of potential confounding factors including age, sex, race, body mass index (BMI), prevalent diabetes, smoking status, estimated glomerular filtration rate (eGFR), factor VIII, von Willebrand factor, antithrombin III, fibrinogen and protein C. Among 14,126 participants, there were 632 incident cases of VTE documented. SUA levels were positively correlated with these cases, even after adjustment for all of the aforementioned factors and independent of other comorbidities or diuretic use. The risk was significantly higher at SUA levels around 8 mg/dL. When analyzing the association of incident gout and VTE, a significant statistical relationship was found only when adjusting for age, sex and race [19]. However, in a large cohort study that included 35,959 patients with previous gout attacks and 35,959 controls that were followed for a mean period of 7.4 years, incident deep vein thrombosis (DVT) was significantly higher in patients with gout, independent of other risk factors for DVT or other confounding factors such as major surgery, pregnancy or anticoagulant treatment [16]. Moreover, in a recently published large population study that included 130,708 patients with gout and 131,349 controls, the risk for VTE events was significantly and independently higher in the gout cohort, both before and after the diagnosis. Furthermore, the risk of incident VTE increased progressively before the gout diagnosis was set, with a peak in the first year prior to diagnosis and in the one after the diagnosis and this association remained strong during 5 years of follow-up. These results remained significant, independent of different covariates represented by medication use; number of hospitalizations and outpatient visits; and comorbidities such as renal failure, hypertension, varicose veins, inflammatory bowel disease, sepsis, and alcohol use [28]. Similarly, in a large cohort that included 62,234 patients with gout and 62,234 controls, the likelihood of the development of DVT was 25% higher after the diagnosis of gout. This association was independent of hospital admissions, urate-lowering therapy or other confounding factors (BMI, smoking status, alcohol use, comorbidities) and remained consistent up to one decade of follow-up [29].

The risk of VTE was also analyzed in a study that included 57,981 patients with gout and 115,961 controls. Once again, patients with gout had a significantly increased risk of VTE, independent of age, sex, fracture of lower limbs, cancer, pregnancy or comorbidities (hypertension, diabetes, AF, dyslipidemia, stroke, heart failure). Moreover, the risk of DVT was higher in gout patients without comorbidities, thus emphasizing the important effect of gout on thrombotic events. In addition, the biggest impact of gout on DVT was in the youngest patients and decreased with advanced age [30].

A significant relationship between SUA levels and the risk of recurrent VTE has been established as well. In their study, De Lucchi et al. included 280 patients with previous VTE and without oral anticoagulant treatment, that were stratified according to SUA tertiles as follows: first tertile ≤ 4.37 mg/dL, second tertile 4.38–5.54 mg/dL, and third tertile ≥ 5.55 mg/dL. The median follow-up period was 71.1 ± 29.2 months. Increased SUA levels were independently associated with a higher risk of VTE recurrence, independent of age, gender, BMI, type of VTE, hypertension, renal function, and previous cardiovascular events. Moreover, the higher the SUA levels, the higher the recurrence risk as SUA levels above 4.38 were associated with more than a three-fold increased risk of VTE recurrence [31].

SUA levels may also be useful in the prognostic stratification of patients with PE. Among 265 patients with unprovoked acute PE, concentrations of uric acid were significantly higher in patients from the intermediate-high to high-risk group according to the European Society of Cardiology (ESC) classification. Moreover, a biomarker model that included uric acid, hemoglobin, and glucose had a prognostic accuracy for adverse outcomes superior to Pulmonary Embolism Severity Index (PESI) and simplified PESI scores and similar to that of ESC classification [32]. In another study that included 337 patients with confirmed PE, SUA values were significantly higher in subjects with intermediate and high risk patients, when compared to low risk patients. Increased levels of SUA were able to predict 30-day mortality in an independent manner. Moreover, higher SUA values were noticed in patients with increased troponin levels and were correlated with the severity of PE [12].

Patients with higher SUA levels that were predisposed to VTE were more likely to be males, with a higher BMI, and a history of diabetes, coronary heart disease, heart failure, hypertension, and renal failure [27,31]. Moreover, the risk of DVT was specifically high for younger patients, with ages below 50 years [29,30].

Table 1 summarizes the included studies about uric acid and VTE.

### 3.2. Uric Acid and Left Atrial Thrombosis

Hyperuricemia was independently associated with an increased risk of both incident and recurrent AF [33,34]. Moreover, different studies analyzed the relationship between SUA levels and the risk of left atrial thrombosis in both AF patients and non-AF patients. Among 284 patients with non-valvular atrial fibrillation (NVAF), SUA was significantly higher in those with associated left atrial thrombosis or spontaneous echo contrast. Moreover, the addition of SUA to CHADS2 and CHA2DS2-VASc scores improved their predictive abilities [35]. Hyperuricemia further increases the risk of thromboembolic events in patients with AF at low-intermediate risk according to CHA2DS2-VASc score [36].

In another study, Liao et al. not only found significantly higher levels of SUA among 1354 patients with NVAF and left atrial appendage spontaneous echo contrast, but even proposed cut-off values points for best predicting it as 371 μmol/L in females and 400 μmol/L in males [18]. In a similar way, Tang et al. proposed cut-off points for SUA best predicting left atrial thrombosis. Among 1359 patients with refractory AF who underwent transesophageal echocardiography (TEE), cut-off points of 359.8 μmol/L in women and 445.6 μmol/L in men were determined by ROC curve. Moreover, when compared to TEE as gold standard, the proposed cut-off values had 76.2% sensitivity and 69.1% specificity in females and 47.5% sensitivity and 77.8% specificity in males. Any other value under the proposed cut-offs had a negative predictive value of 98.1% in women and 97.8% in males [37].

In another study that included 153 patients with AF that underwent TEE, SUA levels were inversely correlated with left atrial appendage flow velocity, thus being capable to independently predict decreased left atrial appendage function, which is known to be correlated with a high prothrombotic state [38].

The association between SUA levels and the risk of left atrial thrombosis was also analyzed in 207 patients in sinus rhythm but with mitral stenosis. Once again, SUA levels were significantly higher in patients with left atrial thrombosis and were positively correlated with inflammatory markers, such as hs-CRP. Moreover, uric acid levels were found to independently predict the risk of left atrial thrombosis among these patients [39].

New evidence in this area was recently published [40] suggesting that not only SUA levels above normal, but also a history of gout/hyperuricemia (considered abnormal uric acid metabolism in general) contributes significantly to the risk of left atrial thrombosis.

Patients prone to left atrial thrombosis were more likely to be older, females, with a greater BMI, and a series of associated comorbidities, such as congestive heart failure, hypertension, vascular disease, previous stroke, diabetes, chronic kidney disease, as well as a persistent/ permanent AF [22,35,37,38,39]. Their biological profile was also particular, with higher mean hemoglobin A1c and higher mean plasma fibrinogen [18,35], and greater hs-CRP levels [37]. The most common echocardiographic peculiarities were represented by larger left atrial dimensions, larger left ventricular end-diastolic and end-systolic diameters, and lower ejection fraction [22,37,39].

In Table 2, we have summarized the main studies about uric acid and left atrial thrombosis.

### 3.3. Uric Acid and MI

In patients with MI, hyperuricemia is nowadays an established risk factor for adverse cardiovascular events, including cardiovascular mortality, independent of the presence of metabolic syndrome [41]. Among 549 patients with acute MI without ST elevation, increased SUA levels were associated with a 1.5-fold higher risk of mortality and with increased in-hospital death rates when compared to normouricemic patients [42], and it seems that the addition of SUA to the GRACE score results in a better prediction of in-hospital mortality among patients with acute coronary syndrome [43]. Furthermore, among 1005 patients with acute MI treated with percutaneous coronary intervention (PCI), preprocedural hyperuricemia was independently associated with increased long-term mortality rates [44].

Recently there were established cut-off values for the optimal prediction of fatal MI and all-cause mortality of 5.7 mg/dL and 4.7 mg/dL respectively [45]. Moreover, increased SUA values were able to independently predict the risk of premature acute MI among 1920 patients [33]. In addition, hyperuricemia may be able to act as a surrogate to unstable coronary plaques [46], thus presenting a very important practical clinical value, as intravascular ultrasonography is not widely available. Increased baseline SUA levels (≥5.4 mg/dL) were also significantly associated with impaired coronary flow after PCI [47], without affecting the risk of periprocedural MI [48].

However, a major limitation of most of the aforementioned results was represented by the evaluation of SUA at a single moment in time as recent studies have emphasized the importance of the changes in SUA levels in time and their relationship with clinical outcomes in patients with MI. In a large prospective study that included 71,449 patients, SUA levels were determined during a 2-year period of follow-up, in order to establish their predictive role in the occurrence of MI. Based on the changes of SUA levels between baseline and follow-up, patients were classified into four categories: stable low, including low SUA levels at baseline and follow-up; elevated, including low SUA levels at baseline and high SUA levels at follow-up; reduced, including high SUA levels at baseline and low SUA levels at follow-up and stable high, including high SUA levels at both baseline and follow-up measurements. Patients with stable high SUA levels were the only ones at high risk of developing MI, with a reported incidence of 2.7 per 1000 person-years [49].

Table 3 summarizes the main studies about uric acid and MI.

### 3.4. Uric Acid and Thromboembolic Risk in Systemic Diseases

SUA levels were correlated with increased risk of thromboembolic events in different systemic disease. Although data on this subject is scarce, we believe it is worth mentioning as it is highly correlated with the topic of this review and may represent a starting point for future investigations. In 173 patients with myelofibrosis, increased SUA values were associated with both worse prognosis and an increased risk of venous and arterial thrombosis [50]. Moreover, in a study that included 93 patients with polycythemia vera and essential thrombocythemia, increased SUA levels were correlated with both a higher risk of thrombosis, as well as with past thrombotic events [51]. Finally, in a study that included 252 patients with Behcet’s disease, among which 50 patients had thrombotic complications, SUA levels were significantly higher in patients with thrombotic complications, when compared to the control group [52].

## 4. Conclusions

Elevated SUA level is associated with a wide range of diseases such as hypertension, chronic kidney disease, heart failure, and coronary artery disease, all these making it an important risk stratification tool. Uric acid has been shown to be a useful biochemical marker of endothelial function, atherosclerosis, development of cardiovascular risk factors, and occurrence of cardiovascular events. In the present review, we discussed its possible involvement in thrombotic events, given the common pathological background with the aforementioned entities. Despite numerous investigations, the causality is still controversial, but it appears that uric acid is a risk predictor for thrombosis and therefore a new potential therapeutic target for its prevention. However, further studies to address the implication of uric acid lowering treatment in reducing the thrombotic risk are needed.

## Figures and Tables

**Table 1:** Characteristics of the clinical studies concerning uric acid and venous thromboembolism.

Study, Year	Study Design	Participants	Outcome, Mean Period of Follow-Up	Results	Conclusions
Kubota Y. et al. [19], 2016	Prospective	14,126 participants aged 45–64, without a history of VTE or gout and not using anticoagulants/gout medications.	VTE occurrence, 22.5 years	632 incident cases of VTE (236 unprovoked and 396 provoked).	Elevated SUA was associated with increased risk of VTE.
Chiu C.C. et al. [16], 2016	Prospective, nationwide longitudinal cohort study	35,959 patients with history of gout attack and 35,959 matched controls without gout.	DVT incidence, 7.4 years	Patients with gout were found to have a 1.38-fold (95% CI, 1.18 to 1.62, *p* < 0.001) higher risk of developing DVT.	The incidence rate of DVT was significantly higher in patients with gout than that in control group.
Li, L. et al. [28], 2020	Prospective, 1:1 matched cohort study	130,708 incident gout patients, and 131,349 non-gout individuals.	First VTE event (either DVT or PE), 15 years	2071 incident VTE, 1377 DVT, and 1012 PE events occurred in the gout cohort, compared with 1629, 1032, and 854 in the non-gout cohort, respectively.	The overall risks of VTE, DVT, and PE were significantly increased both before and after gout diagnosis when compared with the general population.
Sultan, A.A. et al. [29], 2019	Prospective, 1:1 matched cohort study	62,234 patients with incident gout matched to 62,234 controls.	Incident gout cases, 19 years	Gout was associated with higher risk of venous thromboembolism compared with controls (absolute rate 37.3 vs. 27.0)	Gout was associated with higher risk of VTE, particularly when the patient was not in hospital and regardless of exposure to urate-lowering therapy.
Huang, C.C. et al. [30], 2015	Prospective, 2:1 matched cohort study	57,981 patients with gout and 115,961 controls.	Occurrence of VTE (DVT or PE), 12 years	The incidence of DVT was 5.26 per 104 person-years in the gout cohort, which was 2-fold higher than the incidence of 2.63 per 104 person-years in the reference cohort.	Gout increased the risk of DVT and PE.
De Lucchi, L. et al. [31], 2021	Monocenter, prospective study	280 patients with a previous episode of VTE that completed the oral anticoagulant period.	VTE recurrence, 71.1 ± 29.2 months	Patients with SUA levels ≥4.38 mg/dL showed a 3-fold increase in the risk of VTE recurrence.	Elevated SUA levels are associated with increased risk of recurrent VTE independently from traditional risk factors.

VTE, venous thromboembolism; SUA, serum uric acid; DVT, deep vein thrombosis; PE, pulmonary embolism; CI, confidence interval.

**Table 2:** Characteristics of the clinical studies concerning uric acid and left atrial thrombosis.

Study, Year	Study Design	Participants	Outcome	Results	Conclusions
Ning, W. et al. [35], 2017	Retrospective	284 non-valvular AF patients without prior oral anticoagulation.	Additional predictive value of SUA and LAD for CHADS2 and CHA2DS2-VASc.	In 61 patients (21.48%) with LAT/SEC, SUA and LAD were independent risk factors of LAT/SEC and increased the predictive value of CHADS2 and CHA2DS2-VASc.	SUA and LAD enhance the predictive ability of CHADS2 and CHA2DS2-VASc for LAT/SEC.
Numa, S. et al. [36], 2014	Retrospective	470 patients with nonvalvular AF.	Relationship between SUA levels and thromboembolic risk in patients with AF.	SUA level was associated with thromboembolic risk in patients with nonvalvular AF at low-intermediate risk stratified by CHA2DS2-VASc score.	SUA level is an independent predictor of thromboembolic risk on TEE in AF patients at low-intermediate risk.
Liao, H.T. et al. [22], 2015	Retrospective	1476 consecutive hospitalized patients with AF.	Relationship between SUA and LA-SEC in non-valvular AF patients.	SUA level is significantly higher in non-valvular AF patients with LA-SEC.	SUA level is an independent risk factor and has a moderate predictive value for LA-SEC.
Tang, R.B. et al. [37], 2014	Retrospective	1359 consecutive patients undergoing TEE.	Relationship between SUA and the risk of LA thrombus in patients with nonvalvular AF.	SUA levels in patients with LA thrombus were significantly higher.	Hyperuricemia is a risk factor for LA thrombus.
Celik, M. et al. [38], 2015	Retrospective	153 patients with AF who underwent TEE.	Relationship between SUA levels and LAA peak flow velocity.	SUA levels were significant predictors of the LAA peak flow velocity.	High SUA levels are associated with a low contractile function of the LAA.
Ozturk, D. et al. [39], 2015	Retrospective	207 consecutive patients with mitral stenosis who underwent both TTE and TEE.	Risk factors for LA trombus in patients with mitral stenosis in sinus rhythm.	Uric acid was higher in patients with LA thrombus.	A larger LAD and an elevated SUA level are independent predictors of LA thrombosis in patients with mitral stenosis in sinus rhythm.
Zhang, X. et al. [40], 2020	Retrospective	2246 patients who underwent TEE	Risk markers for LA thrombosis.	In 30 patients (1.33%) with LAT, high SUA levels and obesity were risk markers for LAT.	High SUA level is an independent risk marker for LAT. After considering SUA, the CHA2DS2-VASc score for LAT is more accurate.

SUA, serum uric acid; AF, atrial fibrillation; LA, left atrium; LAD, left atrium diameter; LAT/SEC, left atrium thrombosis/ spontaneous echo-contrast; TEE, transesophageal echocardiography; LAA, left atrial appendage; TTE, transthoracic echocardiography.

**Table 3:** Characteristics of the clinical studies concerning uric acid and myocardial infarction.

Study, Year	Study Design	Participants	Outcome	Results	Conclusions
Kuźma, Ł. et al. [42], 2020	Retrospective	549 patients diagnosed with NSTEMI.	Relationship between SUA levels and the long-term prognosis of patients with NSTEMI.	There was a significant correlation between an increase in SUA levels and an increase in mortality (*p* < 0.001).	SUA is an independent risk factor of long-term mortality in patients with NSTEMI, and is associated with higher in-hospital death rates.
Centola, M. et al. [43], 2020	Retrospective	1088 consecutive patients with ACS.	Association between admission SUA levels and in-hospital outcomes in patients with ACS and to investigate the prognostic value of SUA added to GRACE score.	SUA (OR 1.72 95% CI 1.33–2.22, *p* < 0.0001) and GRACE score (OR 1.04 95% CI 1.02–1.06, *p* < 0.0001) were significantly associated with an increased risk of in-hospital death.	High admission levels of SUA are independently associated with in-hospital adverse outcomes and mortality in a population of ACS patients. The inclusion of SUA to GRACE risk score predicts more accurately in-hospital mortality.
Guo, W. et al. [44], 2019	Prospective	1005 AMI patients who underwent PCI.	Prognostic role of hyperuricemia in patients with AMI who underwent PCI.	Mortality for patients with hyperuricemia was higher than that of patients with normal SUA (HR: 1.97; 95% CI: 1.11–3.49; *p* = 0.019).	Preprocedural hyperuricemia is a significant and independent predictor of long-term mortality for patients with AMI who underwent PCI.
Casiglia, E. et al. [45], 2020	Multicentre, observational cohort study	23,467 individuals.	Prognostic cut-off values of SUA in predicting fatal MI.	There was an independent association between SUA and fatal MI in the whole database (HR: 1.381, 95% CI: 1.096–1.758, *p* = 0.006) and in women (HR: 1.514, CI: 1.105–2.075, *p* < 0.01), but not in men.	SUA is an independent risk factor for fatal MI after adjusting for potential confounding variables, and a prognostic cut-off value associated to fatal MI can be identified at least in women.
Xu, J.J. et al. [33], 2020	Prospective cohort study	1920 AMI patients.	Related factors of premature AMI (man ≤ 50 years old, woman ≤ 60 years old).	SUA level (OR = 1.02, 95% CI 1.01–1.04, *p* < 0.01), was an independent related factor of premature AMI.	Metabolic abnormalities, including high SUA, are risk factors of premature AMI.
Saito, Y. et al. [46], 2015	Prospective	81 patients with ACS who underwent intravascular ultrasound-guided PCI.	The relation between SUA level and plaque composition of nonculprit lesions in patients with ACS.	Greater lipid (59.1 ± 9.1% vs. 49.7 ± 10.9% vs. 51.1 ± 9.3%, *p* = 0.001) and less fibrous components (36.8 ± 7.8% vs. 44.3 ± 7.8% vs. 43.2 ± 6.7%, *p* < 0.001) were present in the high than in the low and intermediate SUA levels tertile groups.	Elevated SUA level is associated with greater lipid content of coronary plaque in patients with ACS than in patients with normal levels.
Akpek, M. et al. [47], 2011	Prospective	289 STEMI patients treated with primary PCI.	The association of uric acid levels with coronary blood flow in STEMI.	A uric acid level ≥5.4 mg/dL had a 77% sensitivity and 70% specificity in predicting no-reflow. Uric acid levels (OR 2.75, <95% CI 1.93–3.94; *p* < 0.0001) was an independent predictor of in-hospital MACE.	Plasma uric acid level on admission is a strong and independent predictor of poor coronary blood flow following primary PCI and in hospital MACE among patients with STEMI.
Verdoia, M. et al. [48], 2014	Prospective	1272 consecutive patients undergoing PCI.	The association between SUA levels and periprocedural MI in patients undergoing PCI.	SUA did not affect the risk of periprocedural MI (*p* = 0.29; adjusted OR = 1.11 [0.93–1.32], *p* = 0.26) or periprocedural myonecrosis (*p* = 0.97; adjusted OR = 0.99 [0.86–1.14], *p* = 0.89).	SUA is not associated with an increase in the risk of periprocedural MI in patients undergoing percutaneous coronary revascularization.
Tian, X. et al. [49], 2020	Prospective	71,449 Chinese participants.	The association between both baseline SUA and changes in SUA and the risk of MI.	In 837 MI cases identified during follow-up, MI risk was only associated with stable high SUA (HR: 1.42 95% CI: 1.02–1.92, *p* = 0.03), compared with those with stable low SUA. There was no association between hyperuricemia at baseline and MI (HR 1.14, 95% CI: 0.91–1.42, *p* = 0.19).	Only stable high SUA is associated with increased higher risk of MI. Changes in SUA levels in any other direction or high SUA levels at baseline were not associated with risk of MI.

SUA, serum uric acid; NSTEMI, non-ST-elevation myocardial infarction; STEMI, ST-elevation myocardial infarction; ACS, acute coronary syndrome; AMI, acute myocardial infarction; MI, myocardial infarction; PCI, percutaneous coronary intervention; MACE, major adverse cardiac events; HR, hazard ratio; CI, confidence interval; OR, odds ratio.

## Data Availability

Not applicable.
